# Differentiation of C_4_ photosynthesis along a leaf developmental gradient in two *Cleome* species having different forms of Kranz anatomy

**DOI:** 10.1093/jxb/eru042

**Published:** 2014-02-18

**Authors:** Nuria K. Koteyeva, Elena V. Voznesenskaya, Asaph B. Cousins, Gerald E. Edwards

**Affiliations:** ^1^Laboratory of Anatomy and Morphology, V. L. Komarov Botanical Institute of Russian Academy of Sciences, Prof. Popov Street 2, 197376, St. Petersburg, Russia; ^2^School of Biological Sciences, Washington State University, Pullman, WA 99164-4236USA

**Keywords:** C_4_ anatomy, C_4_ development, *Cleome angustifolia*, *Cleome gynandra*, C_4_ photosynthesis, C_4_ plants, immunolocalization, ultrastructure.

## Abstract

In family Cleomaceae there are NAD-malic enzyme-type C_4_ species having different forms of leaf anatomy. Leaves of *Cleome angustifolia* have Glossocardioid-type anatomy with a single complex Kranz unit which surrounds all the veins, while *C. gynandra* has Atriplicoid anatomy with multiple Kranz units, each surrounding an individual vein. Biochemical and ultrastructural differentiation of mesophyll (M) and bundle sheath (BS) cells were studied along a developmental gradient, from the leaf base (youngest) to the tip (mature). Initially, there is cell-specific expression of certain photosynthetic enzymes, which subsequently increase along with structural differentiation. At the base of the leaf, following division of ground tissue to form M and BS cells which are structurally similar, there is selective localization of Rubisco and glycine decarboxylase to BS cells. Thus, a biochemical C_3_ default stage, with Rubisco expression in both cell types, does not occur. Additionally, phosphoenolpyruvate carboxylase (PEPC) is selectively expressed in M cells near the base. Surprisingly, in both species, an additional layer of spongy M cells on the abaxial side of the leaf has the same differentiation with PEPC, even though it is not in contact with BS cells. During development along the longitudinal gradient there is structural differentiation of the cells, chloroplasts, and mitochondria, resulting in complete formation of Kranz anatomy. In both species, development of the C_4_ system occurs similarly, irrespective of having very different types of Kranz anatomy, different ontogenetic origins of BS and M, and independent evolutionary origins of C_4_ photosynthesis.

## Summary

In *Cleome* species (Cleomaceae) having two different forms and evolutionary origins of Kranz anatomy there is convergence in the structural and biochemical expression of C_4_ traits during leaf ontogeny.

## Introduction

In most C_4_ plants, the dual-cell system (so-called Kranz anatomy) operates to concentrate CO_2_ around ribulose 1,5-bisphosphate carboxylase-oxygenase (Rubisco). Primary fixation of CO_2_ into C_4_ acids occurs in the outer layer of mesophyll (M) cells using phosphoenolpyruvate carboxylase (PEPC), while decarboxylation of C_4_ acids and refixation of CO_2_ by Rubisco in the C_3_ pathway occur in the inner layer of bundle sheath (BS) cells (Hatch, 1987). This CO_2_-concentrating mechanism restricts the competing ribulose bisphosphate (RuBP) oxygenase reaction and synthesis of glycolate, and the loss of CO_2_ by photorespiration. Glycine decarboxylase (GDC), which is essential for photorespiratory CO_2_ release in the glycolate pathway, is confined exclusively to BS mitochondria (Edwards and Walker, 1983; Edwards and Voznesenskaya, 2011; Sage *et al.*, 2012).

C_4_ species are classified according to their biochemical subtype based on the predominant type of C_4_ decarboxylase, NADP-malic enzyme (NADP-ME), NAD-malic enzyme (NAD-ME), or phosphoenolpyruvate carboxykinase, used to release and concentrate CO_2_ around Rubisco (Hatch, 1987). The two main types of C_4_ cycles in eudicots function either through NADP-ME, which is located in the chloroplasts, or through NAD-ME, which is located in the mitochondria of BS cells. There are structural differences in grana development between M and BS chloroplasts in NADP-ME- versus NAD-ME-type C_4_ species associated with differences in photochemistry. For C_4_ to function, NADPH is required to reduce 3-phosphoglycerate, the product of CO_2_ fixation by Rubisco, to triose-phosphates. In NADP-ME-type C_4_ species, the primary shuttle of malate from M cells to BS chloroplasts provides both CO_2_ and NADPH, which reduces the requirement for photosystem II (PSII) activity, and the associated development of grana, in BS chloroplasts. In NAD-ME-type species, BS chloroplasts have increased grana development and PSII activity, since the primary shuttle of aspartate from the M to BS cells delivers CO_2_, but not reductive power (Edwards and Walker, 1983; Edwards and Voznesenskaya, 2011).

C_4_ photosynthesis is considered to have evolved independently >60 times (Sage *et al.*, 2011), resulting in much structural diversity in the forms of C_4_ which evolved from C_3_ ancestors (Dengler and Nelson, 1999; Peter and Katinas, 2003; Muhaidat *et al.*, 2007; Edwards and Voznesenskaya, 2011). Among the forms identified, 16 develop Kranz around individual veins, while eight develop a compound Kranz unit near the periphery of the leaf, which surrounds all veins (Edwards and Voznesenskaya, 2011). The diversity in forms occurs by special positioning of the M and BS layers in relation to the distribution of veins and other tissues, by the occurrence of different types of tissues in the leaf (e.g. mestome sheath in monocots, hypodermal, or water storage cells in dicots), by the way organelles (chloroplast and mitochondria) are arranged in BS cells and their differentiation between M and BS cells, and by structural diversity in developing gas diffusion barriers to support the CO_2_-concentrating mechanism (Edwards and Voznesenskaya, 2011).

The ontogeny of the complex C_4_ system requires a highly coordinated selective expression of many genes during development, concurrent with establishment of Kranz anatomy. Genes controlling development of M and BS specialization for function of the C_4_ system, and the degree of redundancy which has occurred during the multiple times C_4_ has evolved is unknown. Among monocot and eudicot C_4_ species which are being used as genetic models, concerted efforts are needed to identify regulatory factors along leaf developmental gradients which control the expression of specific C_4_ traits (Covshoff *et al.*, 2013).

There have been a number of studies on structural and biochemical development of forms of C_4_ having Kranz anatomy around individual veins. This includes extensive analysis of leaf development in *Zea mays* which has classical NADP-ME-type anatomy (Sheen and Bogorad, 1985; Langdale *et al.*, 1988b; Dengler and Nelson, 1999; Sheen, 1999). In the interest of identifying transcription factors controlling expression of specific traits which are required for C_4_ function, there have been recent studies on the developmental gradient along the leaf of maize, including analyses of transcriptome, proteome, and structural changes (see Li *et al.*, 2010; Majeran *et al.*, 2010; Covshoff *et al.*, 2013; Wang *et al.*, 2013). Developmental studies have also been conducted on some other forms of Kranz anatomy in grasses (Dengler *et al.*, 1985, 1986, 1996; Wakayama *et al.*, 2003) and on different structural forms of Kranz in family Cyperaceae (Soros and Dengler, 2001). Among eudicots, significant developmental studies have been conducted on two NAD-ME-type species having Atriplicoid-type anatomy, namely *Atriplex rosea* (Liu and Dengler, 1994; Dengler *et al.*, 1995; Dengler and Nelson, 1999) and *Amaranthus hypochondriacus* (Ramsperger *et al.*, 1996; Patel and Berry, 2008). There has been less attention to development of types of Kranz anatomy which surround all veins in the leaves. There are reports on NADP-ME Salsoloid type with *Salsola richteri* (Voznesenskaya *et al.*, 2003b), NAD-ME Salsinoid type with *Suaeda taxifolia*, and NAD-ME Schoberioid type with *Suaeda eltonica* (Koteyeva *et al.*, 2011*a*).

These studies on different forms of C_4_ show a progression in structural and biochemical differentiation during development, with dimorphic M and BS cells originating from monomorphic cells. Characterization of the development of Kranz anatomy includes identification of the origins of M and BS cells during leaf initiation, which is different depending on Kranz anatomical type or plant lineages (Dengler *et al.*, 1985; Dengler and Nelson, 1999; Soros and Dengler, 2001; Koteyeva *et al.*, 2011*a*). This characterization includes structural differentiation of M and BS chloroplasts and their function in photochemistry to provide energy cooperatively for CO_2_ assimilation (Brown, 1975; Dengler *et al.*, 1985; Langdale *et al.*, 1988*a*, *b*; Dengler and Nelson, 1999; Sheen, 1999; von Caemmerer and Furbank, 2003; Patel and Berry, 2008; Li *et al.*, 2010; Majeran *et al.*, 2010; Edwards and Voznesenskaya, 2011; Koteyeva *et al.*, 2011*a*; Wang *et al.*, 2013). It also consists of analysis of the differences among forms of C_4_ in the development of barriers to diffusion of CO_2_ from sites of decarboxylation in BS cells, which enables CO_2_ to be concentrated around Rubisco (e.g. BS cell wall thickness, location of the C_4_ acid decarboxylase, and the position of organelles) (von Caemmerer and Furbank, 2003). Determining when selective expression of enzymes in M and BS cells for C_4_ function occurs during development among different forms of C_4_ is also important (Dengler *et al.*, 1985; Soros and Dengler, 2001; Koteyeva *et al.*, 2011*a*; Langdale, 2012). Analyses show that there are differences in the sequence and the timing of biochemical and structural changes, with respect to establishment of the vascular system, and whether expression of traits is regulated by environmental (light) or developmental cues (Langdale *et al.*, 1988*a*; Wang *et al.*, 1992; Dengler *et al.*, 1995; Ramsperger *et al.*, 1996; Dengler and Nelson, 1999; Sheen, 1999; Voznesenskaya *et al.*, 2003*a*; Wakayama *et al.*, 2003; Koteyeva *et al.*, 2011*a*; Langdale, 2012).

There is recent interest in the occurrence of C_4_ photosynthesis in Cleomaceae, one of 19 families in which C_4_ species have been found (Sage *et al.*, 2011). The genus *Cleome sensu lato* consists of >200 species. Most species have the C_3_ type of photosynthesis; but, it has been shown that three species, *Cleome angustifolia*, *C. gynandra*, and *C. oxalidea*, have NAD-ME-type C_4_ photosynthesis in leaves and cotyledons (Marshall *et al.*, 2007; Voznesenskaya *et al.*, 2007; Feodorova *et al.*, 2010; Koteyeva *et al.*, 2011b). Two of them, *C. gynandra* and *C. oxalidea*, have flat leaves with an Atriplicoid type of anatomy consisting of multiple Kranz units around individual veins, while *C. angustifolia* with Glossocardioid-type anatomy, has a single compound Kranz unit with a double layer of concentric chlorenchyma surrounding all the veins and water storage cells (Koteyeva *et al.*, 2011b). They belong to separate lineages in the genus and they have different distribution, pantropical for *C. gynandra*, Australian for *C. oxalidea*, and African for *C. angustifolia* (Feodorova *et al.*, 2010). *Cleome gynandra* was originally proposed as a model NAD-ME eudicot C_4_ species for identifying factors controlling development and function of C_4_ photosynthesis (Brown *et al.*, 2005). The attractive features of this species are that it can be transformed, grown under a short life cycle, has C_3_ and C_3_–C_4_ intermediate *Cleome* relatives for comparative studies, and, phylogenetically, among C_4_ genera it has the closest position to the well-established model species *Arabidopsis thaliana* (a C_3_ dicot) (Feodorova *et al.*, 2010; Covshoff *et al.*, 2013). Progress towards development of *C. gynandra* has been made, including identifying transcripts associated with C_4_ biochemistry and metabolite transporters (Kajala *et al.*, 2012), in comparative RNaseq profiling (Bräutigam *et al.*, 2011), and in the ability to transform it (Newell *et al.*, 2010).

The purpose of the current study was to examine development of C_4_ in two NAD-ME-type C_4_ *Cleome* species, *C. gynandra* and *C. angustifolia*, which have major differences in leaf morphology, vascular pattern, and forms of Kranz anatomy. This included analysis of M and BS cell formation from leaf primordia, and structural and biochemical transitions during leaf ontogeny, to determine whether there is convergence in development and expression of C_4_ traits.

## Materials and methods

### Plant material

Seeds of *Cleome angustifolia* Forssk. (from herbarium material kindly provided by Drs A. Oskolskii and O. Maurin) and *C. gynandra* L. (kindly provided by Dr A.S. Raghavendra) were stored at 3–5 ºC prior to use and were germinated on moist paper in Petri dishes at room temperature and a photosynthetic photon flux density ~20 μmol quanta m^–2^ s^–1^. The seedlings were then transplanted to 15cm diameter pots with commercial potting soil and grown in the greenhouse during the mid-winter/spring months, with an approximate 26 ºC day/18 ºC night temperatures. Maximum midday photosynthetic photon flux density was 500 μmol quanta m^–2^ s^–1^ on clear days. Plants were fertilized once a week with Peter’s Professional (20:20:20; Scotts Miracle-Gro Co., Marysville, OH, USA). For microscopy and biochemical analyses, leaves of different lengths were taken from plants of ~3–6 weeks old. Voucher specimens are available at the Marion Ownbey Herbarium, Washington State University under # WS 375818 for *C. angustifolia* and WS 369765 for *C. gynandra.*

### Light and electron microscopy

Study of the structural basis of leaf development was carried out on expanding leaves with lengths of leaflets from 0.5mm to 7mm. Vegetative shoot tips were used to study the earliest events of leaf initiation and development. Three samples of each leaflet length (using the terminal leaflet in the palmate leaf) were harvested from three independent plants of each species (a total of nine samples from each species for each leaf length). Vegetative shoot apices with several of the youngest leaf primordia, and leaves of intermediate size (6–7mm in length), were sectioned longitudinally. Additionally, cross-sections were made in 0.5mm steps from the tip to base of 3–5mm long leaves.

Sample preparation for light microscopy (LM) and transmission electron microscopy (TEM) was carried out following Koteyeva *et al.* (2011*a*). An Olympus BH-2 (Olympus Optical Co. Ltd) light microscope equipped with an LM Digital Camera and Software (Jenoptik ProgRes Camera, C12plus, Jena, Germany) was used for observation and collection of images at the LM level. A Hitachi H-600 (Hitachi Scientific Instruments, Tokyo, Japan) and FEI Tecnai G2 (Field Emission Instruments Company, Hillsboro, OR, USA) equipped with Eagle FP 5271/82 4K HR200KV digital camera transmission electron microscopes were used for TEM studies.

The cell size, length of the chloroplasts, and the thickness of cell walls were measured for the adaxial M and BS on micrographs from leaf longitudinal and cross-sections at different distances from the leaf base using an image analysis program (UTHSCSA, Image Tool for Windows, version 3.00). For both types of cells, the measurements of cell wall thickness were made on cell walls facing intercellular airspaces between M and BS. From images of the cell walls, plasmodesmata between M and BS cells (which were radially but not longitudinally oriented in the wall sections) were counted. The plasmodesmata frequency was referred to as the number of plasmodesmata per 1 μm of cell–cell contact interface length.

Images of the shoot apices with the youngest leaf primordia and surface of young leaves for analysis of stomata were captured under the low vacuum mode with an FEI Scanning Electron Microscope Quanta 200F (Feild Emission Instruments Company) without additional treatments.

To observe the leaf vascular pattern, leaves of different ages, from youngest primordia to fully expanded leaves, were cleared in 70% ethanol (v/v) until chlorophyll was removed, bleached with 5% (w/v) NaOH overnight, and then rinsed three times in water. At least five leaves of different ages were studied from two to three different plants. The leaves were mounted in water on slides and examined under a UV light [with a 4′,6-diamidino-2-phenylindole (DAPI) filter] on a Fluorescence Microscope Leica DMFSA (Leica Microsystems Wetzlar GmbH, Germany) using autofluorescence of lignified tracheary elements of the xylem.

### *In situ* immunolocalization

Sample preparation and immunolocalization by LM and TEM were carried out on longitudinal sections of leaves, 6–7mm long, following the procedure in Koteyeva *et al.* (2011*a*). Antibodies used were anti-spinach Rubisco [large subunit (LSU)] IgG (courtesy of B. McFadden) commercially available anti-maize PEPC IgG (Chemicon, Temecula, CA, USA), and the P-subunit of GDC, all raised in rabbits. The density of labelling was determined by counting the gold particles on digital electron micrographs using the UTHSCSA image analysis program and calculating the number per unit area (μm^2^). Standard errors were determined.

### Western blot analysis

For the study of accumulation of the main photosynthetic enzymes during development, individual leaflets of different ages, with lengths of 1–3, 3–6, 6–10, 10–15mm, and 30–35mm (totally expanded mature leaflet), were used. Soluble proteins were extracted from leaves and prepared for SDS–PAGE following Koteyeva *et al.* (2011b). Protein concentration was determined with an RCDC protein quantification kit (Bio-Rad), which tolerates detergents and reducing agents. A 10 μg aliquot of soluble protein was applied per lane, with separation by 12% (w/v) SDS–PAGE, and transferred to a nitrocellulose membrane for analysis of the several photosynthetic enzymes and GDC. Primary antibodies used were anti-*Amaranthus hypochondriacus* NAD-ME IgG against the 65kDa α-subunit (Long and Berry, 1996) (dilution 1:5000), anti-*Zea mays* PEPC IgG (1:100 000), anti-*Zea mays* pyruvate,Pi dikinase (PPDK) IgG (courtesy of T. Sugiyama) (1:5000), anti-*Pisum sativum* L. GDC P-subunit (courtesy of Dr D. Oliver) (1:10 000), and anti-*Spinacia oleracea* Rubisco LSU IgG (courtesy of B. McFadden) (1:10 000). Goat anti-rabbit IgG–alkaline phosphatase-conjugated secondary antibodies (Sigma) were used at a dilution of 1:10 000 for detection. Bound antibodies were visualized by developing the blots with 20mM nitroblue tetrazolium and 75mM 5-bromo-4-chloro-3-indolyl phosphate in detection buffer (100mM TRIS-HCl, pH 9.5, 100mM NaCl, and 5mM MgCl_2_). Two separate blots from two separate extractions were made for each enzyme. The intensities of bands in western blots were quantified with an image analysis program (ImageJ 1.37, NIH, USA) and expressed relative to the level in the fully expanded leaves.

### Mass spectrometric measurements of Γ, *R*_d_, and leaf carbon isotope composition

A membrane inlet mass spectrometer (DELTA V Plus; Thermo Scientific) connected to a closed leaf chamber via a membrane inlet was used to measure rates of CO_2_ exchange and the CO_2_ compensation point (Γ) in *C. gynandra* young and mature leaves, as described previously (Maxwell *et al.*, 1998; Ruuska *et al.*, 2000). Cuttings from mature leaves ~1.5cm^2^ or 5–7 young (~7mm) leaves of *C. gynandra* harvested from several branches were placed in the chamber and, once CO_2_ and O_2_ concentrations were balanced with ambient concentrations, the leaf chamber was sealed and after 5min sitting in the dark was submitted to an irradiance of 1000 μmol m^–2^ s^–1^. The temperature was controlled at 25 °C using a water bath circulating around the chamber. Net CO_2_ assimilation was followed in the sealed chamber by measuring the changes in ^12^CO_2_ concentration over time until reaching Γ; that is, when the amount of CO_2_ assimilated by photosynthesis was balanced with the amount of CO_2_ released by respiration and photorespiration. The rate of dark respiration (*R*_d_) was calculated from the rate of CO_2_ released after a minimum of 5min in the dark. The system was zeroed before and after each measurement by flushing the chamber with a mixture of nitrogen and oxygen.

Leaf carbon isotope composition was determined in *C. gynandra* from sections taken at the base, middle, and tip of young 0.7cm leaves, and from mature leaves (*n*=3–6). As previously described (Voznesenskaya *et al.*, 2013), samples (1–2mg) were placed in a tin capsule and combusted in a Eurovector elemental analyser; the resulting N_2_ and CO_2_ gases were separated by gas chromatography and admitted into the inlet of a Micromass, Isoprime isotope ratio mass spectrometer (IRMS) for determination of ^13^C/^12^C ratios (R). δ^13^C values were determined where 1000×R_sample_/R_standard_)–1, using PDB (Pee Dee Belemnite) as the standard.

### Statistical analysis

Where indicated, standard errors were determined, and analysis of variance (ANOVA) was performed with Statistica 7.0 software (StatSoft, Inc.). Tukey’s HSD (honest significant difference) tests were used to analyse differences in cell and chloroplast sizes, cell wall thickness, plasmodesmata frequency, amounts of gold particles, intensities of bands in western blots, and Г and *R*_d_ values at different stages of leaf development. All analyses were performed at the 95% significance level.

## Results

### Early events in primary leaf morphogenesis

**Fig. 1.**
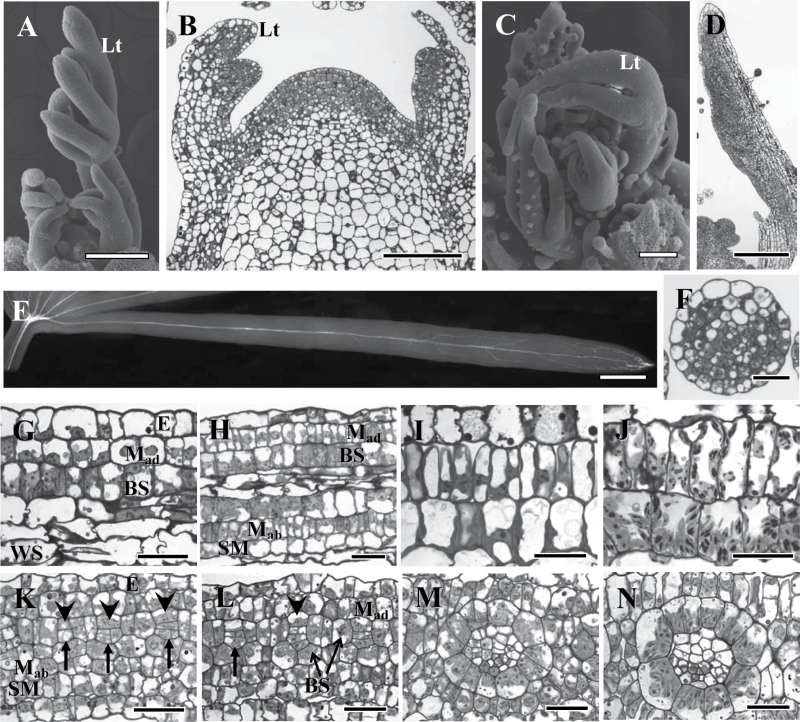
Vegetative shoot tip structure and early stages of leaf development in two *Cleome* species, *C. angustifolia* (A, B, E–J) and *C. gynandra* (C, D, K–N). (A, C) Scanning electron microscopy of shoot tips. (B, D) Light microscopy of longitudinal sections of the shoot apical meristem showing initiation and development of the leaflet. (E) Cleared young leaflet of *C. angustifolia* under UV light showing the basipetal direction of lateral vein development. (F) Cross-section of the young (0.5mm) *C. angustifolia* leaf. (G–J) Longitudinal sections showing the formation of cell lineages from the base of young leaves (G) to the tip (J), with the direction of maturation from left to right in *C. angustifolia.* The adaxial double-layered chlorenchyma (M and BS) differentiation is shown at Stages 1 (G), 2 (H, note the abaxial SM delimited), 3 (I), and 4 (J). (K–N) Procambial strand initiation and Kranz anatomy development around the veins from the base of young leaves (K) to the tip (N), with the direction of maturation from left to right in *C. gynandra*. (K) Procambium initials are indicated by arrows; arrowheads show the first adaxial BS cell progenitor; note the additional abaxial SM layer formed. (L) BS progenitors derived from second and third ground meristem layers are indicated. Different stages of M and BS differentiation are shown for *C. gynandra*: Stage 1 (L), 2 (M), and 3 (N). E, epidermis; BS, bundle sheath; Lt, leaflet; M_ad_, adaxial mesophyll; M_ab_, abaxial mesophyll; SM, spongy mesophyll; WS, water storage. Scale bars=200 μm for A, C, D; 100 μm for B; 0.5mm for E; 20 μm for F–N.

*Cleome angustifolia* and *C. gynandra* have palmate compound leaves with up to five flat, broad leaflets in *C. gynandra* and 5–8 narrow leaflets in *C. angustifolia*. Figure 1 shows the morphology and anatomy of a vegetative shoot apical meristem of *C. angustifolia* and *C. gynandra* at the stage of active organogenesis, together with the early stages of morphogenesis of compound leaves (Fig. 1A–D). Leaf primordia in both species are initiated alternately. When the primordium reaches ~100 μm, the proliferation of marginal and submarginal initials appears at the distal adaxial flank (Fig. 1B), giving rise to the terminal followed by the lateral primordial leaflets (Fig. 1A, C). Leaflets of *C. angustifolia* grow by meristem initials at the base generating files of cells with restricted marginal growth resulting in narrow leaflets. Laminar growth in the leaflets of *C. gynandra* is initiated when they reach 0.4–0.5mm long, forming a flat blade (Fig. 1D).

In both species, the processes of tissue differentiation in terminal and lateral leaflets are similar; but they differ in growth dynamics, resulting in different sizes (Fig. 1A, C). Procambial strands of the future major veins are initiated acropetally (developing from the base towards the tip) in leaflets. From analysis of cleared young leaves of different ages, the visualization of lignified elements of xylem confirms an acropetal pattern of differentiation of major veins, and shows a basipetal pattern of peripheral/minor vasculature development. In *C. angustifolia*, the lateral veins develop from the tip (Fig. 1E). In general, *C. gynandra* also has a basipetal pattern of secondary vascular system development from the tip of the leaf and the leaf margins to the base; however, veins of different orders have different time courses of initiation, with later development of the higher order veins (not shown).

Leaflet development in compound leaves does not differ from that in simple leaves, as shown earlier for different species (Esau, 1965). The leaflet of *Cleome* species will be referred to later as ‘leaf’ for simplicity in presentation.

### Ontogenetic origin of mesophyll and bundle sheath cells

The origin of M and BS cells was observed in cross- and longitudinal sections at the base of young leaves (leaf length of 0.5–5mm for *C. angustifolia* and 3–7mm for *C. gynandra*). In *C. angustifolia*, M and BS cell precursors originate during the initiation of leaf primordium from the ground meristem, with subsequent division of abaxial M progenitors which give rise to the M layer adjacent to BS cells and an additional layer of abaxial chlorenchyma [precursors of the spongy mesophyll (SM) cell layer]. The middle layers of ground meristem generate water storage tissue and procambial strands. Young leaf primordia (up to 0.5mm) consist of 6–7 layers of ground meristem and the forming central procambial strand (excluding epidermis, Fig. 1F). During the subsequent leaf blade development, the M and BS cells divide only anticlinally, and the developing leaf consists of 7–8 cell layers between epidermises depending on the number of progenitors of water storage cells (Fig. 1H).

In *C. gynandra*, the youngest leaves (up to 3mm long) and the base of young leaves (7mm long) consist of four layers of ground meristem between the adaxial and abaxial epidermis (not shown), with the abaxial subepidermal cells undergoing periclinal divisions very early in development and forming an additional abaxial layer which will later become SM (Fig. 1K, L). Periclinal division of the ground meristem cells in the second layer leads to initiation of the procambial strand from the abaxial derivative (Fig. 1K, arrows show the different stages of procambial strand formation). The adaxial derivative is the progenitor of the upper BS cell (or 2–4 upper BS cells in the case of subsequent anticlinal divisions; Fig. 1K, arrowheads show the upper BS progenitors). All other BS cells originate from the second and third layers of ground meristem which surround the forming vascular tissues (Fig. 1L). The M is differentiated from the first, second, third, or fourth layer of the ground meristem cells depending on the position of M cells around the ring (on the cross-section) of BS cells.

### Development of Kranz anatomy

The formation of Kranz anatomy was studied at the LM and TEM levels using longitudinal sections of intermediate size young leaves. Clear gradual progression in distinct developmental events can be observed along the leaf from the base to the tip. In both species, the meristematic activity is evident near the base of the leaf, followed by BS cell expansion with M cell intensive divisions, resulting in 2–3M cells per BS cell as viewed on the leaf section. In both species the additional abaxial layer of SM cells has not undergone anticlinal divisions, in contrast to their sister abaxial M (M_ab_) cells which are adjacent to the BS (Fig. 1H, L). After transition of M cells to expansion, the structural differentiation of M and BS cells begins, and close to the leaf tip complete Kranz anatomy is established. Following the initial formation of M and BS cells from progenitor cells, four stages of chlorenchyma development were characterized in the progression towards the formation of the Kranz syndrome.

#### Stages of chlorenchyma development in *C. angustifolia*

Development was visualized using files of cells which were continuous from the base to the tip of the 6mm long leaf. In Stage 1 at the meristematic zone of the base of the leaf (0–1mm from the base), M and BS cells are similar in shape, size, and structure (Fig. 1G; Table 1). They can be identified by their position, from the adaxial side of the leaf subepidermal layer for M, and BS for the next layer, with an additional subepidermal layer of SM cells on the abaxial side. At this time M and BS cells undergo anticlinal divisions.

**Table 1.** Cell size (length, L, and width, W), thickness of cell walls, size of chloroplasts for mesophyll (M) and bundle sheath (BS) cells, and the number of plasmodesmata in the cell wall between M and BS cells

Stage of development	Cell size (μm)				Thickness of individual cell wall towards the IAS (μm)		No. of plasmodesmata per μm M/BS cell wall	Chloroplast length (μm)	
	M		BS		M	BS		M	BS
	L	W	L	W					
*Cleome angustifolia*									
1	12.5±0.5 a	9.1±0.5 b	12.5±0.3 a	9.7±0.7 a	ND	ND	ND	1.5±0.07 a	1.6±0.06 a
2	15.4±0.2 b	5.4±0.3 a	16.4±0.4 b	11.1±0.6 a	0.12±0.009 a	0.23±0.01 a	0.53±0.12 a	1.5±0.08 a	2.3±0.1 a
3	20.3±0.2 c	4.9±0.2 a	19.6±0.5 b	13.1±0.4 b	0.12±0.02 a	0.27±0.01 b	1.25±0.16 b	2.3±0.08 b	3.0±0.08 b
4	35.6±1.0 d	8.5±0.4 b	27.1±0.7 c	15.6±1.1 c	0.14±0.006 a	0.28±0.01 b	1.1±0.14 b	5.6±0.2 c	4.8±0.2 c
*Cleome gynandra*									
1	10.3±0.3 a	6.6±0.3 a	9.7±0.3 a	10.8±0.6 a	ND	ND	ND	2.5±0.1 a	2.4±0.1 a
2	16.0±0.6 b	6.7±0.3 a	13.6±0.5 b	15.3±0.9 b	0.08±0.01 a	0.21±0.02 a	1.11±0.09 a	3.5±0.2 a	3.8±0.2 b
3	19.1±0.6 c	6.4±0.2 a	15.7±0.5 b	15.1±1.0 b	0.10±0.01 a	0.30±0.02 b	0.88±0.17 a	4.6±0.1 b	5.1±0.2 c
4	21.5±0.6 d	6.5±0.2 a	18.8±0.6 c	17.9±0.8 b	0.10±0.006 a	0.43±0.02 c	0.81±0.09 a	5.6±0.2 c	7.1±0.2 d

The average number of partial cell profiles examined was 20.

Different letters indicate significant differences between developmental stages in a column for each species separately, at *P*≤0.05.

**Fig. 2.**
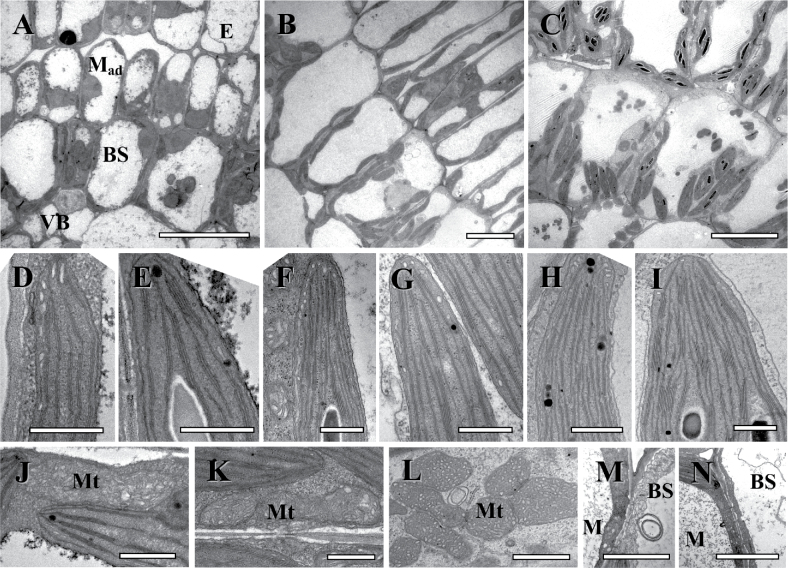
Electron microscopy of *Cleome angustifolia* longitudinal sections showing three structural stages along the leaf from the base to the tip with the undifferentiated cells at the base, developmental Stage 2 (A, D, E, J), through Stage 3 (B, F, G, K, M), and nearly mature cell structure at the tip, Stage 4 (C, H, I, L, N). (A–C) Micrographs show the development of leaf structure and organelle distribution in BS and M_ad_ cells. (D, F, G) Thylakoid system in M chloroplasts. (E, G, I) Thylakoid system in BS chloroplasts. (J–L) Progression in BS mitochondria ultrastructure specialization and size increase. Note the specific cristae in mitochondria in mature BS cells. (M, N) Plasmodesmata between M and BS cells and BS cell wall thickening. E, epidermis; BS, bundle sheath; M, mesophyll; M_ad_, adaxial mesophyll; Mt, mitochondria; VB, vascular bundle; WS, water storage tissue. Scale bars=10 μm for A–C; 0.5 μm for D–K; 1 μm for L; 2 μm for M, N.

Following the completion of division of M cells, the expansion of M and BS cells is evident (~2–3mm from the base) (Table 1), and this was designated as Stage 2. At this time M and BS cells have a central vacuole, and chloroplasts are distributed around the cell periphery. In M cells, the nucleus often has a central position (Fig. 2A). Chloroplasts have a relatively well-developed thylakoid system with numerous small grana which are structurally similar in both M and BS cells (Fig. 2D, E). Mitochondria in the chlorenchyma cells are few in number, small, and they have crescent-like cristae (Fig. 2J). The cells of all tissues are tightly packed; minor intercellular air spaces (IAS) exist between M and epidermal cells (Fig. 2A). Xylem and phloem elements are not differentiated in veins.

Stage 3 (~3–5.5mm from the base) of chlorenchyma development is characterized by structural differentiation of M and BS. M cells have developed a palisade shape with a well-developed central vacuole and organelles distributed around the cell periphery (Figs 1I, 2B). The characteristic centripetal positioning of organelles in BS cells is established (Fig. 1I, Fig. 2B). BS and M chloroplasts are structurally similar; but, compared with Stage 2, they have become enlarged (Table 1) and have a more developed thylakoid system (Fig. 2F, G). BS mitochondria are co-localized with chloroplasts, and they are also slightly enlarged (Fig. 2K). Intercellular air spaces throughout the M cell layer are more prominent (Figs 1I, 2B). The numbers of plasmodesmata connecting M and BS cells have increased (Fig. 2M; Table 1). At this stage, there are the first signs of vascular tissue differentiation in the minor veins. In most vascular bundles, 1–2 tracheary elements are well developed, while the sieve elements of the phloem are still not completely differentiated as they contain a cytoplasmic layer with organelles delimited by the tonoplast. Mature xylem vessels can be identified by the prominent secondary wall thickening and the lack of cytoplasm. Mature sieve tubes contain only a peripheral layer of cytosol without nuclei, ribosomes, and tonoplasts, with rare occurrence of mitochondria, plastids, and smooth endoplasmic reticulum (not shown).

In Stage 4, observed in the leaf tip (~5.5–6mm from the base), differentiation of chloroplasts and mitochondria is completed and there is full formation of Glossocardioid-type Kranz anatomy. Chloroplasts of BS cells have numerous grana with 5–15 thylakoids in stacks, while M chloroplasts have poorly developed grana usually with 2–3 thylakoids in stacks (Fig. 2H, I). BS mitochondria are larger and more numerous compared with Stage 3, with cristae showing a distinct tubular structure (Fig. 2L). BS organelles maintain their characteristic positioning in the cell. Also, multiple plasmodesmata connect BS cells with M, as was noted in Stage 3 (Fig. 2N; Table 1). Between Stages 2 and 4, there is not a significant difference in the thickness of the BS cell wall towards the IAS; however, the BS cell walls are about twice as thick as those in M cells (Table 1).

#### Stages of chlorenchyma development in *C. gynandra*

Compared with *C. angustifolia*, in *C. gynandra* the basal meristematic zone is extended, with delimitation of BS cells occurring after 1mm. Development of Kranz anatomy was followed in cross-sections of veins of the highest orders (4 and 5, minor veins) along the longitudinal section from the base, where cell proliferation occurs, to the tip where BS and M cells are differentiated. While in general the veins differentiate basipetally, the differentiation of neighbouring veins alternates according to their order and timing of initiation, with corresponding changes in development of BS cells. On the other hand, M cells have a clear longitudinal developmental gradient from the base to the tip of the leaf, which is independent of the level of vein and BS differentiation.

At the earliest stages near the base of the leaf, the BS cells are identified by their position adjacent to developing vein tissues, and subsequently by their size and roundish shape (Fig. 1K–N). In Stage 1 (~1–3mm from the base), anticlinal and radial cell divisions occur in M and BS cells; the two types of cells have nearly the same appearance, with the BS cells being a little wider (Fig. 1L; Table 1). Vascular bundles are not differentiated and they appear as multiplying procambium cells (Fig. 1L).

**Fig. 3.**
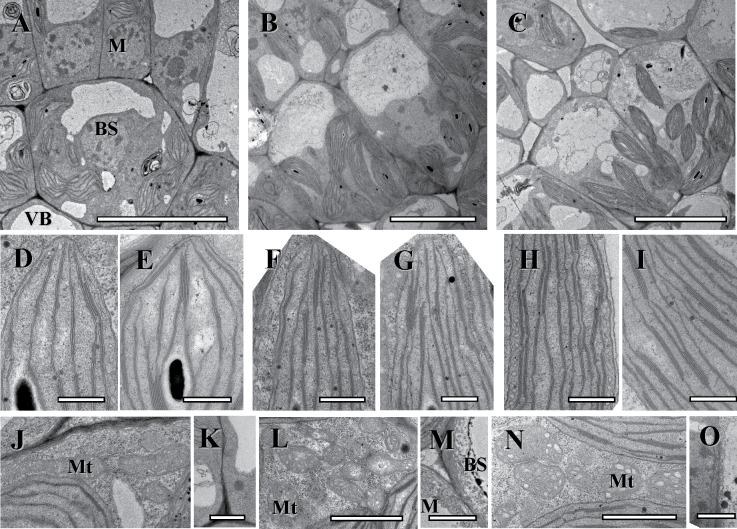
Electron microscopy of *Cleome gynandra* longitudinal sections showing three structural stages along the leaf from the base to the tip, with the undifferentiated cells at the base, Stage 2 (A, D, E, J, K), through Stage 3 (B, F, G, L, M), and nearly mature cell structure at the tip, Stage 4 (C, H, I, N, O). (A–C) Micrographs show the development of leaf structure and organelle distribution in BS cells. (D, F, H) Thylakoid system in M chloroplasts. (E, G, I) Thylakoid system in BS chloroplasts. (J, L, N) Progression in BS mitochondria ultrastructure specialization and size increase. Note the specific cristae in mitochondria in mature BS cells. (K, M, O) Plasmodesmata between M and BS cells and BS cell wall thickening. BS, bundle sheath; M_ad_, adaxial mesophyll; Mt, mitochondria; VB, vascular bundle. Scale bars=10 μm for A–C; 0.5 μm for D–I; 1 μm for J–O.

Recognition of Stage 2 in *C. gynandra* is associated structurally with transition of M cells from division to expansion (~4–5.5mm from the base). BS cells of the highest order veins are characterized by the vacuoles which are asymmetrically forming at the centrifugal pole (Figs 1M, 3A) which ultimately results in the shift of organelles to the centripetal position in Stage 3. As in *C. angustifolia*, at this stage of development BS and M chloroplasts are enlarged compared with Stage 1 (Table 1) and they have a similar thylakoid system with numerous small grana (Fig. 3D, E). The mitochondria are small with crescent-like cristae (Fig. 3J for BS cell). Most leaf tissues are tightly packed. The BS cell wall is about twice as thick as the M cell wall, and there are multiple plasmodesmata connecting BS and M cells (Table 1). In veins, there is the beginning of differentiation of xylem and phloem elements.

At Stage 3 of development (~5.5–6.5mm from the base), M and BS cell structural specialization becomes clearer, with well-developed central vacuoles in the M cells and distinctive centripetal organelle positioning in BS cells (Figs 1N, 3B). Chloroplasts of both cell types have increased in size (Table 1) and they have a similar level of grana development (Fig. 3F, G). Bundle sheath mitochondria have increased in number and are localized towards the inner cell wall (Fig. 3L). At this stage, the BS cell walls have continued to thicken and there is no change in plasmodesmata number per unit of cell wall length between the M and BS (Fig. 3M; Table 1). Also, differentiation of 2–3 xylem tracheary elements and first phloem sieve elements has occurred at this stage of development.

In Stage 4, at the leaf tip (~6.5–7mm from the base), the Atriplicoid type of Kranz anatomy is fully developed. The size of M and BS cells and chloroplasts increased (Table 1). The ultrastructural dimorphism of the chloroplast and mitochondria has formed. Chloroplasts of M cells usually have 2–3 thylakoids in grana stacks, while BS chloroplasts have numerous grana with 4–10 thylakoids in stacks (Fig. 2H, I). BS cells have abundant, large mitochondria with distinct tubular cristae (Fig. 3N). The BS cell wall facing the IAS is four times thicker than in M; the numbers of plasmodesmata between the M and BS are similar to those in Stage 3 (Fig. 3O; Table 1).

Stomatal initiation from epidermal cells occurred along the longitudinal leaf gradient, with a clear progression in the portion of fully differentiated stomata from the base to mid-region to the tip which was similar for the two species (8, 26, and 72%, respectively, in *C. angustifolia* and 5, 33, and 69%, respectively in *C. gynandra*) (Supplementary Fig. S1 available at *JXB* online). Supplementary Fig. S1 shows images of the abaxial surface of the leaves (results were similar for the adaxial surface, not shown).

### Immunolabelling along the developmental gradient

The pattern of PEPC, Rubisco LSU, and GDC expression was studied on longitudinal sections of intermediate size leaves (~6–7mm) using the reflected/scanning mode of confocal microscopy. The more precise immunogold technique by TEM was used to analyse the base, mid-region, and tip of the same leaf to evaluate the density of labelling in cellular compartments.

**Fig. 4.**
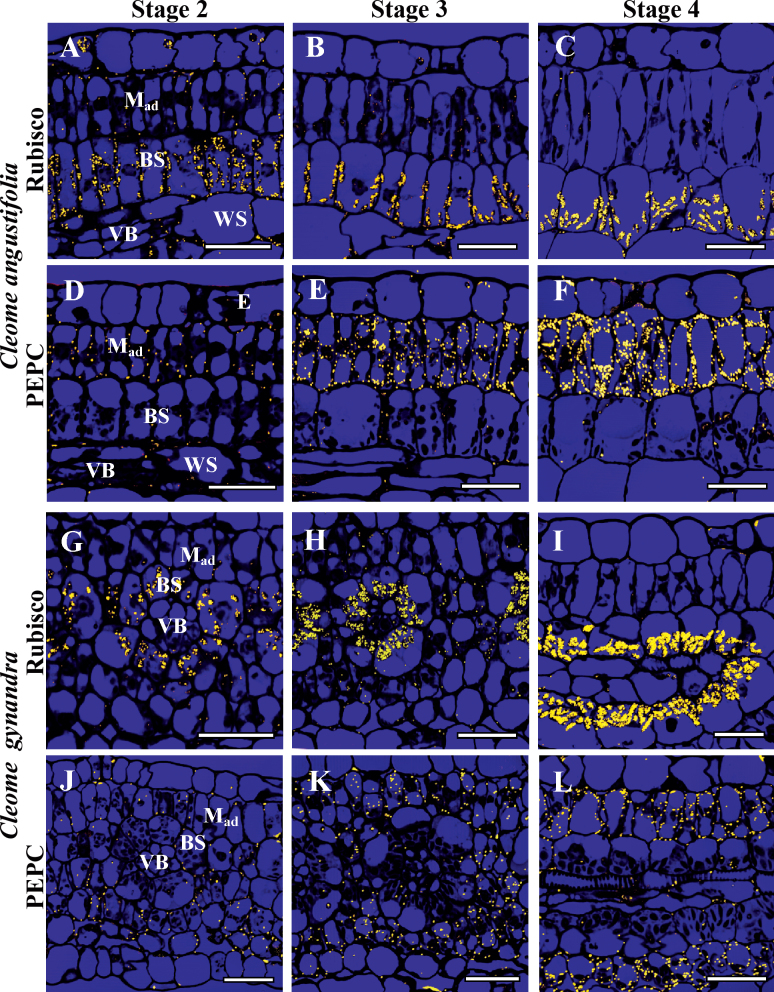
*In situ* immunolocalization of Rubisco LSU (A–C, G–I) and PEPC (D–F, J–L) in leaves of *Cleome angustifolia* (A–F) and *C. gynandra* (G–L) at three developmental stages. Reflected/transmitted confocal imaging of longitudinal leaf sections shows Rubisco LSU selectively localized to BS cells beginning from developmental Stage 2 (A, G); note labelling in the few chloroplasts in the epidermis and water storage cells (A). The weak labelling for PEPC is M specific from the earliest stages (D, J), with increased labelling beginning from Stage 3 (E, K). At Stage 4, high labelling for Rubisco and PEPC is partitioned to BS and M, respectively. BS, bundle sheath; E, epidermis; M_ad_, adaxial mesophyll; VB, vascular bundle, WS, water storage. Scale bars=20 μm for A–L.

**Fig. 5.**
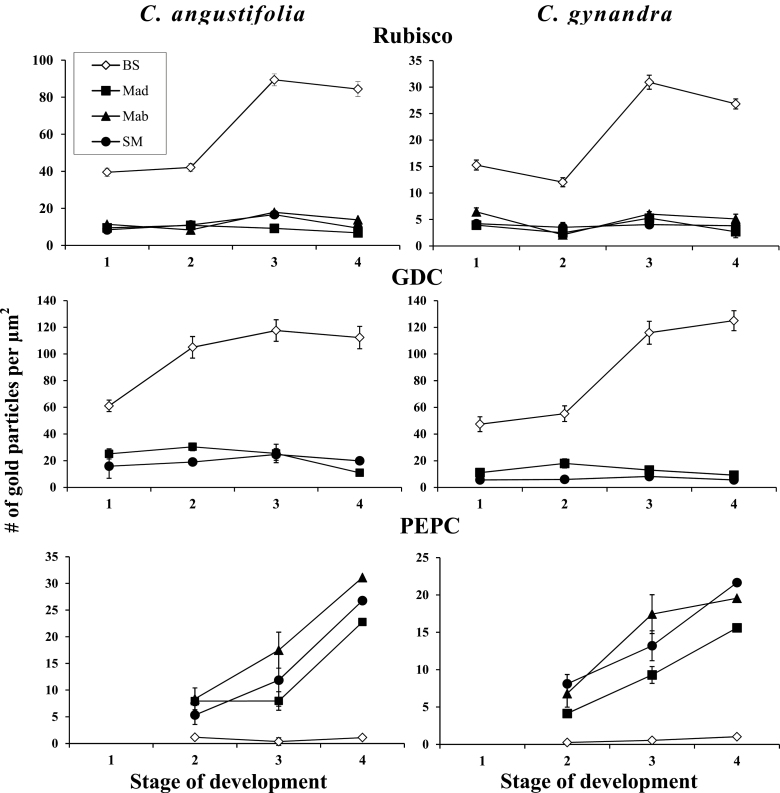
Graphical representation showing the density of immunolabelling for Rubisco LSU (top panels) in BS versus M chloroplasts, GDC (middle panels) in BS versus M mitochondria, and PEPC (bottom panel) in BS versus M cytosol at four stages of leaf development for *Cleome angustifolia* and *C. gynandra.* Also, the comparison was made between three types of M cells: M_ad_, adaxial mesophyll; M_ab_, abaxial mesophyll cells which are in contact with the BS cells; and SM (spongy mesophyll), an additional M layer on the abaxial side of the leaf which does not have contact with BS. In both graphs, the *y*-axis represents the number of gold particles per μm^2^ of chloroplast, mitochondria, or cytosol area, and the *x*-axis represents the developmental stages. For each cell type and stage of development, 10–15 cell areas were used for counting. BS, bundle sheath; M, mesophyll.

In both *Cleome* species, Rubisco is strongly associated with BS chloroplasts from Stage 1 when M and BS cells become distinguishable (Figs 4A, C, 5). Rubisco is suppressed not only in chloroplasts in M cells adjacent to BS (M_ad_ and M_ab_), but also in the SM cells which are not in contact with the BS cells (Figs 4G, H, 5). Rubisco accumulation per BS chloroplast area reaches a maximum level in Stage 3 (Figs 4B, H, 5), and maintains a high level in Stage 4 at the tip of leaves (Figs 4C, I, 5).

In both species there is selective labelling of GDC in BS mitochondria in Stage 1. In *C. angustifolia* GDC expression increases and reaches a maximum at Stage 2 of development, which is well before BS cell differentiation and mitochondrial structural specialization. In *C. gynandra* a steady increase of GDC was shown in parallel with an increase in BS Rubisco levels (Fig. 5) and cell structural maturation.

PEPC was not detected by immunolocalization in Stage 1 (not shown). In Stage 2, at the confocal microscopy level, there is very low M-specific labelling for PEPC in both *Cleome* species (Fig. 4D, J). The TEM level shows a low and comparable amount of PEPC selectively localized in M_ad_ cells and in both M_ab_ and SM cells (Fig. 5). Both methods show steady accumulation of PEPC preferentially in M cells, with levels reaching a maximum at the tip of the leaf (Stage 4) with little or no difference in particle density between M_ab_ cells adjacent to BS and the SM layers (Figs 4E, F, K, L, 5). Compared with the M_ad_ cells, the expression of PEPC begins earlier in the M_ab_ and SM cells, and usually the density of labelling is higher in these cells at all stages of leaf development (Fig. 5). For *C. gynandra*, comparative counting of the number of particles in the M cells located around the neighbouring veins of different order (with a different level of BS differentiation) shows no difference (not shown).

### Western blot analysis

**Fig. 6.**
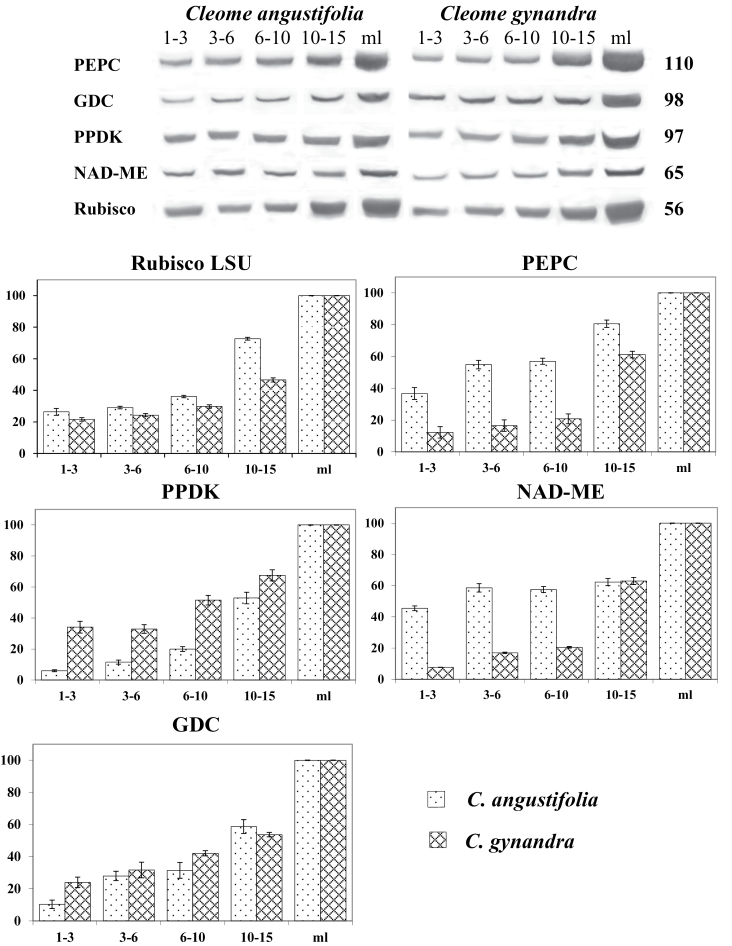
Western blot analysis showing accumulation of C_4_ enzymes, GDC, and Rubisco LSU during leaf development in two *Cleome* species. Total soluble proteins were extracted from individual leaves of different lengths of *C. angustifolia* and *C. gynandra*. Blots were probed with antibodies raised against PEPC, GDC, PPDK, NAD-ME, and Rubisco LSU. Top: representative western blots showing detection of each protein. Numbers on the right indicate molecular mass in kilodaltons. Bottom: quantitative representation of western blot data. 100% on the *y*-axis refers to the level achieved in mature leaves. The *x*-axis represents the length of the leaves.

Western blots were performed on extractions of total soluble proteins from individual leaves (young leaves of different lengths, 1–3, 3–6, 6–10, and 10–15mm, and mature leaves, 30–35mm) (Fig. 6). Proteins for the C_3_ pathway (Rubisco), C_4_ cycle (PEPC, PPDK, and NAD-ME), and photorespiratory pathway (GDC) were analysed and represented quantitatively (on a soluble protein basis). The youngest leaves (1–3mm) of both *Cleome* species contain 22–26% of Rubisco (LSU) compared with the mature stage, with a slow increase in leaves up to 6–10mm long and a large increase thereafter. The youngest leaves (1–3mm) of *C. gynandra* contain a low amount of C_4_ enzymes; only 12% of PEPC, 6% of PPDK, and 8% of NAD-ME compared with mature leaves on a soluble protein basis; there was a slow rise in levels in leaves up to 10mm long, and a large increase thereafter. In contrast, the youngest leaves of *C. angustifolia* have a high amount of C_4_ enzymes which on average is 40% of that in the mature stage, with increasing levels as leaf age progressed. The level of GDC shows a steady increase from the youngest up to mature leaves, with higher expression in *C. gynandra* than in *C. angustifolia* (Fig. 6).

### CO_2_ compensation point, dark respiration, and carbon isotope composition in *C. gynandra*

**Fig. 7.**
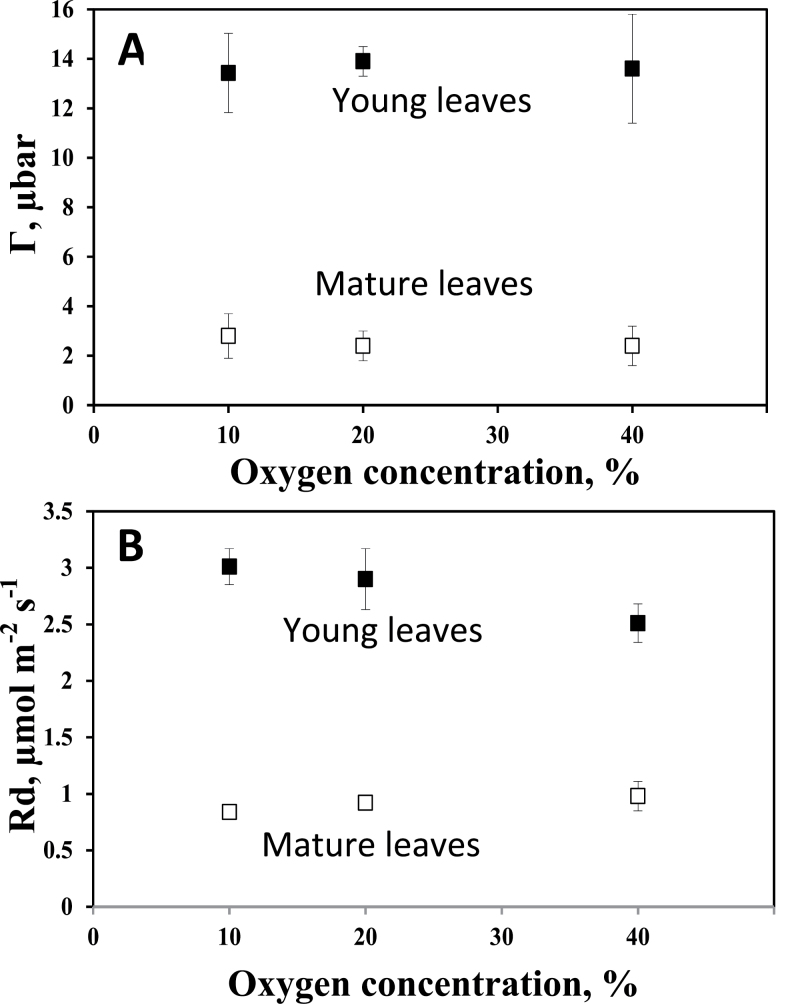
The effect of oxygen on the CO_2_ compensation point (Γ; A) and dark respiration (*R*_d_; B) in young and mature leaves of *Cleome gynandra.* Number of replications *n*=5–8 for young leaves and *n*=3–6 for mature leaves. One-way analysis of variance shows that varying O_2_ had no significant effect on Γ or *R*_d_ in young or mature leaves.

Г and *R*_d_ were measured at 10, 20, and 40% O_2_ in young (~7mm) and mature leaves of *C. gynandra* (Fig. 7). Г was insensitive to increases in O_2_ in both; however, the mean value of Г across all O_2_ levels was higher in the young (13.7±1.7 μbar) than in mature leaves (2.6±0.8 μbar). Rates of *R*_d_ were higher in young than in mature leaves, with mean values across O_2_ levels of 2.91±0.33 in young versus 0.91±0.08 μmol m^–2^ s^–1^ in mature leaves. Excised leaves of *C. angustifolia* were unstable in the leaf chamber, so it was not possible to obtain steady-state readings with the mass spectrometer. Values of carbon isotope composition (δ^13^C) measured from leaf sections of *C. gynandra* at the base, tip, and middle of young leaves were –14.1 (±0.03), –14.1 (±0.02), and –14.7 (±0.08), respectively, compared with –14.6 (±0.12) in mature leaves.

## Discussion

### Origin of M and BS cells in C_4_ *Cleome*

*Cleome angustifolia* and *C. gynandra* have compound leaves with a similar pattern of initiation, but with distinct differences in the patterns of minor veins and Kranz anatomy spatial development. In *C. angustifolia*, which has a form of Kranz (called Glossocardioid) that consists of a single complex unit, M and BS cells originate from subepidermal layers of ground meristem during leaflet initiation. The pattern of origins of M and BS cells is very close to that in *S. taxifolia* which has Schoberioid-type anatomy with a similar positioning of two chlorenchyma layers at the leaf periphery (Koteyeva *et al.*, 2011*a*). In *C. gynandra*, which has Atriplicoid-type anatomy with Kranz units around individual veins, the BS cells towards the adaxial side are sister to procambium cells arising from the adaxial derivative of the first periclinal division of the ground meristem cell (the abaxial derivative gives rise to the procambium initial). The rest of the BS cells ontogenetically originate from the ground meristem cells of the second, third, and fourth layers adjacent to the vein. M cells are also derived from the ground meristem having different ontogeny, namely subepidermal and central cells, which is similar to that of *A. rosea* with Atriplicoid-type anatomy (Liu and Dengler, 1994). In both *C. angustifolia* and *C. gynandra* it is difficult to reveal the progenitor cells of the shoot apical meristem due to initiation of leaflets from the primary primordium which is the future petiole. As shown in previous studies, the ontogenetic origin of M and BS cells has no effect on the structural, biochemical, and functional peculiarities of C_4_ tissues which shows a clear convergent evolution of a highly coordinated functioning of the two-cell system (Dengler *et al.*, 1985, 1986, 1996; Koteyeva *et al.*, 2011*a*).

### Longitudinal patterns of development in C_4_ *Cleome*

The form of Kranz in leaves of *C. angustifolia* allows all stages of the developmental progression to be studied along one file of chlorenchyma cells from the base to the tip of an intermediate size leaf. A similar basipetal pattern of development along a single lineage of cells has also been observed in two other forms having a single compound Kranz unit (Schoberioid and Salsinoid type in family Chenopodiaceae, Koteyeva *et al.*, 2011*a*), and in several C_4_ monocots with parallel venation (Langdale *et al.*, 1988*a*; Soros and Dengler, 2001; Wakayama *et al.*, 2003; Li *et al.*, 2010; Majeran *et al.*, 2010; Nelson, 2011).

In contrast to *C. angustifolia*, development of Kranz anatomy in leaves of *C. gynandra* is closely associated with vein differentiation. Having in general a longitudinal basipetal pattern, the initiation of minor veins is sequential in time. Veins at different stages of differentiation in *C. gynandra*, which are alternate in the longitudinal section, can be easily recognized by size, anatomy, chloroplast ultrastructure, and accumulation of Rubisco in the BS cells. The pattern of vascular development in *C. gynandra*, and associated progression in specialization of BS cells, resembles that described for C_4_ *A. rosea* (Dengler *et al.*, 1995), C_4_ *A. hypochondriacus* (Wang *et al.*, 1993), and C_4_ *Flaveria trinervia* (McKown and Dengler, 2009).

### Stages of C_4_ development along the leaf

**Fig. 8.**
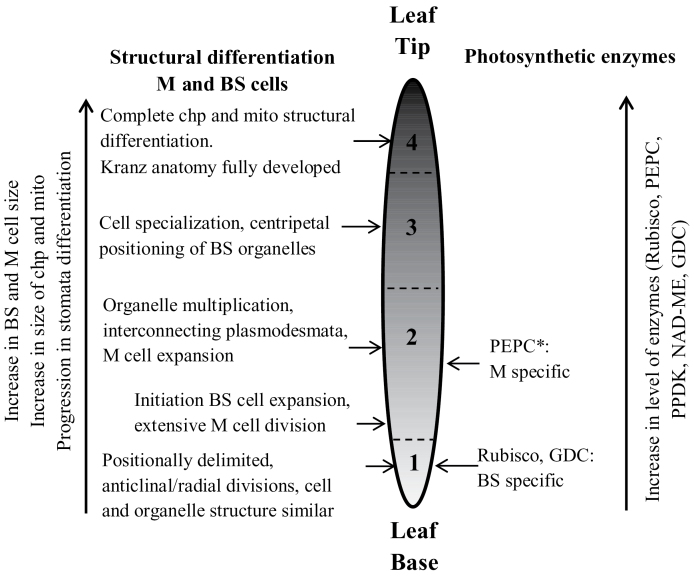
Illustration of major changes during development of C_4_ photosynthesis in *Cleome angustifolia* and *C. gynandra* from the basal region to the tip of young leaves. The numbers refer to four stages of chlorenchyma development; see text for the corresponding length of the zones for each species. The horizontal arrows point to specific changes occurring in each of the four stages. The vertical arrows indicate continual changes which occur along the longitudinal gradient. BS, bundle sheath, chp, chloroplasts; GDC, glycine decarboxylase; M, mesophyll; mito, mitochondria. *PEPC was selectively localized to M cells in stage 2 (there was insufficient labelling to determine its localization by immunolocalization in stage 1).

Irrespective of having vastly different Kranz anatomical types, *C. angustifolia* and *C. gynandra* have a similar sequence of events during C_4_ leaf development. However, the lengths of the zones along the leaves where transitions occur are different between the two species, which may be related to differences in anatomy or growth rates. Four notable stages in C_4_ development were recognized from base to tip of young leaves of both species based on anatomical and ultrastructural features (illustrated in Fig. 8).

#### Structural development

Stage 1, the beginning of Kranz, is distinguished by M and BS cells which are positionally delimited from meristem precursors, performing anticlinal/radial divisions and having similar shape and structure with ultrastructurally uniform organelles. In Stage 2 there is expansion of M and BS cells, vacuoles are developing, and chloroplasts and mitochondria are replicating and enlarging. The plasmodesmata frequency between M and BS cells is also high at this time. In Stage 3, structural specialization of M and BS begins. The M cells develop a palisade-like shape with a large central vacuole and organelles around the cell periphery. Organelles in BS cells become localized in a centripetal position towards the inner cell wall characteristic of mature Kranz anatomy in C_4_ *Cleome* species. The chloroplasts, and the mitochondria, between M and BS cells are structurally similar, indicating an immature stage of development. In Stage 4, full Kranz anatomy has developed, with structurally specialized dimorphic chloroplasts and mitochondria in M and BS cells.

There were some structural parameters which show a continuous change along the leaf gradient. In both species there was an increase in the sizes of M and BS cells, chloroplasts, and mitochondria, and in stomatal development and density (illustrated on the left side of Fig. 8). There was an increase in the length of the M cells with no change in width, while there was an ~2-fold increase in both the length and width of the BS cells. During development there is continual enlargement of M and BS chloroplasts, with an increase in length by ~3-fold in both species. The density of plasmodesmata at the interphase between M and BS cells increases or remains constant (see Table 1) despite the increase in size of M and BS cells (and contact between the two) due to the formation of secondary plasmodesmata during development. This indicates establishment of symplastic connections between M and BS from the earliest stages of development which enables intercellular movement of metabolites for C_4_ photosynthesis. In both species, BS cell wall thickening, which starts soon after the BS cell become positionally delimited, is considered to contribute to diffusive resistance and limit CO_2_ leakage from the BS during C_4_ photosynthesis (von Caemmerer and Furbank, 2003).

#### Biochemical development

In both species there is a very early cell-specific C_4_-like pattern of enzyme accumulation. BS cells complete divisions, and initiate cell expansion with multiplication of chloroplasts and mitochondria earlier than M cells which continue to have active anticlinal divisions up to Stage 2. However, biochemical specialization in M and BS begins simultaneously from the meristematic zone in Stage 1. Rubisco and GDC are selectively expressed in chloroplasts and mitochondria, respectively, in BS cells, and at the same time they are suppressed in chloroplasts and mitochondria of the M cells, characteristic of the C_4_ system. So, while organelles, chloroplasts and mitochondria, do not become ultrastructurally differentiated in BS and M until full maturation of Kranz, biochemical specialization of organelles in the two chlorenchyma cells is occurring already in the meristematic stages right after the layers become positionally delimited. This shows that Rubisco and GDC expression in these two *Cleome* species is not directly associated with structural differentiation of M and BS cells.

Early cell-specific Rubisco expression during leaf development has also been observed in several other C_4_ species, including *Arundinella hirta* (Wakayama *et al.*, 2003), *A. rosea* (Dengler *et al.*, 1995), *S. eltonica*, and *S. taxifolia* (Koteyeva *et al.*, 2011*a*). In contrast, a C_3_ default state with Rubisco appearing initially in both M and BS chloroplasts has been observed in other C_4_ species, with selective expression of Rubisco in BS depending on light or developmental cues in maize (Sheen and Bogorad, 1985; Langdale *et al.*, 1988b; Sheen, 1999), in *A. hypochondriacus* (Patel and Berry, 2008), in three structural forms of C_4_ in family Cyperaceae (Soros and Dengler, 2001), and in *Salsola richteri* (Voznesenskaya *et al.*, 2003b). Clearly these differences in transitions to C_4_ suggest evolution of alternative cues for initiation of C_4_ development; although there may be convergence in factors (transcriptional or transacting) controlling synthesis of components of the C_4_ system.

PEPC levels were too low to detect in Stage 1. From its earliest detection in Stage 2, it was M specific and the level increased in parallel with M differentiation. In a number of other developmental studies, PEPC gene expression also appears to be initially M specific and increased depending on M differentiation (Langdale *et al.*, 1988*a*; Dengler *et al.*, 1995; Stockhaus *et al.*, 1997; Soros and Dengler, 2001; Wakayama *et al.*, 2003; Koteyeva *et al.*, 2011*a*).

The results in both C_4_ *Cleome* species indicate that intercellular compartmentation for C_4_ photosynthesis is established early without a C_3_ default state, that enzyme levels to support the C_4_ system continue to rise during the four stages of leaf development, and that structural positioning of organelles and differentiation of chloroplasts and mitochondria occur later (Fig. 8). This suggests that development of C_4_ biochemistry may be a driver for organelle differentiation, namely for M and BS chloroplasts to provide the balance in ATP and NADPH from photochemistry to support the NAD-ME-type C_4_ cycle (see Edwards and Voznesenskaya, 2011), and for mitochondria to provide the capacity for transport and metabolism to support decarboxylation via NAD-ME. The same pattern of development in these two C_4_ *Cleome* species which have very different types of Kranz anatomy and independent evolutionary lineages is consistent with the concept that genes to support the C_4_ cycle were already primed for recruitment and selective expression in M and BS cells (Brown *et al.*, 2011; Kajala *et al.*, 2012).

A functional analysis of young versus mature leaves of *C. gynandra* was made by analysing the effect of O_2_ on Г to determine if the response is indicative of C_4_, intermediate, or C_3_ photosynthesis. C_4_ plants have very low Г (1–5 μbar) while C_3_ plants have high values of Г (~40–50 μbar at 25 ^○^C and 20% O_2_). Additionally, Г in C_4_ plants has little or no sensitivity to O_2_, while values in C_3_ plants approximately double with a 2-fold increases in O_2_. C_3_–C_4_ species have intermediate Г values, which increase with the level of O_2_ (Krenzer *et al.*, 1975; Brooks and Farquhar, 1985; von Caemmerer, 1989, 2000; Ku *et al.*, 1991; Vogan *et al.*, 2007; Voznesenskaya *et al.*, 2007). Mature leaves of *C. gynandra* show a clear C_4_-type response, with values of Г of ~2.5 μbar with insensitivity to O_2_. In young leaves, the lack of sensitivity of Г to O_2_, between 10% and 40%, is characteristic of C_4_ plants. The higher Г values in young leaves (~14 μbar) are probably due to the higher *R*_d_ and lower *V*_cmax_ for Rubisco (see western blots), due to the dependence of Г on the *R*_d_/*V*_cmax_ ratio (von Caemmerer, 2000), in addition to its dependence on Rubisco kinetic properties and the level of photorespiration. In plant leaves, *R*_d_ saturates at relatively low levels of O_2_ (~2–3% O_2_), whereas the reaction of O_2_ with RuBP oxygenase, in competition with CO_2_, results in an increase in Г in response to higher levels of O_2_. Thus, in the absence of *R*_d_, extrapolated plots of Г versus O_2_ would intercept at zero, whereas contribution from *R*_d_ results in the intercept on the *y*-axis (Brooks and Farquhar, 1985). Thus, the results suggest that young leaves of *C. gynandra* are functionally C_4_. A factor which may minimize the occurrence of photorespiration and maintain a lower Г in young leaves is the selective localization of photosynthetic enzymes to M and BS cells for function of C_4_ early in development. Also, the selective localization of GDC to the BS indicates the presence of conditions favourable for refixation of photorespired CO_2_ around Rubisco from the very early stages of development. As leaves develop, there is an increase in the level of GDC in the two *Cleome* species; this may be associated with multiplication and increase in size of BS mitochondria (where GDC is localized), which was observed during development along a longitudinal gradient in young leaves (Stage 3). There is the possibility that sensitivity of photosynthesis to O_2_ occurs at the base of young leaves, but its contribution to carbon assimilation is expected to be very low (due to low Rubisco, and undeveloped stomata).

### Expression of C_4_ enzymes in M cells which do not have contact with the BS

Both *Cleome* species have an additional layer of chlorenchyma cells on the abaxial side of leaves (SM) which is ontogenetically related to the abaxial M layer which is adjacent to the BS. They are not associated with BS, and structurally resemble M cells with well-developed chloroplasts. It was shown by confocal immunohistochemistry and TEM immunogold labelling that their cytosol contains PEPC at a similar level to that of adaxial and abaxial M cells which are in contact with BS. The expression of Rubisco and GDC in these distal M cells is suppressed, and structural and biochemical differentiation of these cells occurs in parallel with the M cells in contact with BS.

There are not many cases where C_4_ plants have more than two M cells between neighbouring BS cells; analysis of 119 C_4_ grasses (Hattersley and Watson, 1975) showed that no M cell is separated from the nearest BS cell by more than one other M cell (the maximum cell distant count). Based on studies with maize, it has long been considered that M cells differentiate to form a C_4_-type cell only when they are in contact with BS; and that M cells distant from BS are deprived of PEPC expression and contain Rubisco in chloroplasts (Langdale *et al.*, 1988b). In maize husk having >20 additional cells between BS cells, the structural and biochemical specialization for C_4_ was shown only in M cells which are in contact with BS cells, which supports this hypothesis (Langdale *et al.*, 1988b; Pengelly *et al.*, 2011). Also, in *A. rosea* there is an extra layer of abaxial and adaxial cells which are not in contact with the BS cells; Dengler *et al.* (1995) found that PEPC expression was restricted to the M cells adjacent to BS cells along the whole maturation gradient. However, the authors noted that those cells contain few organelles and are non-photosynthetic (hypodermal cells, probably functioning in water storage). Thus, the results with the two C_4_ *Cleome* species, which clearly shows formation of C_4_-type M cells which are not adjacent to the BS, is an interesting variation on regulation of C_4_ development.

### Signalling for tissue-specific development of BS and M cells

Veins in C_4_ plants have been considered to play an organizing role in the differentiation of Kranz anatomy as a source of the positional signal which controls the coordinated development of at least two cells in a radius around individual veins, as in *C. gynandra*, generating the pattern of Vein–BS–M–M–BS–Vein units (Langdale *et al.*, 1988*a*, *b*; Nelson and Langdale, 1992; Langdale, 2012) and candidate genes which may be associated with differentiation of Kranz anatomy have been proposed (Wang *et al.*, 2013). This is a logical hypothesis for the leaf structures where the anatomy consists of multiple simple Kranz units around individual veins which occur in many C_4_ lineages coupled with increased leaf venation during evolution from C_3_ to C_4_ species (McKown and Dengler, 2007; Griffiths *et al.*, 2013). However, the pattern of development of Kranz anatomy in the *Cleome* species in the current study is not compatible with this hypothesis. In *C. gynandra*, which has Atriplicoid-type anatomy, the structural development of M specialization is not correlated with BS cell development. Rather BS cell development is correlated with stages of differentiation of alternate veins, but not the M cells which have only a basipetal gradient of maturation. Also, the hypothesis of signalling from vascular tissue to control differentiation of the M cells adjacent to BS cells is not consistent with the same expression pattern of C_4_ in the cells of the abaxial SM layer not adjacent to BS. These two abaxial M layers represent sister cells which originated from the division of the abaxial subepidermal ground meristem; but, their segregation occurs before, or simultaneously with, the start of procambial strand initiation. One could imagine that the ‘M’ signal may come to the progenitor cell before its division, which could explain the existence of PEPC in both M layers; but, in this case, the signal cannot come from the undifferentiated vein. Also, the selective expression of PEPC in the M cells occurs later, during Stage 2, while the selective expression of Rubisco and GDC occurs during Stage 1, which suggests temporal differences in signalling.

With respect to *C. angustifolia*, recently it was shown that evolution of C_4_ species having a single compound Kranz unit in C_4_ *Salsola* occurs not with increasing vein density, but by increased succulence and development of a layer of BS cells around veins and water storage tissue (Voznesenskaya *et al.*, 2013). For differentiation of tissue for this type of anatomy, veins could not be the centre for a positional signal since they are not evenly distributed relative to the position of Kranz anatomy, and their contact with BS is not essential (Koteyeva *et al.*, 2011*a*). The spatial positioning of M and BS cells relative to each other, unlike the positioning of Kranz around individual veins, is essential to the mechanism controlling their differentiation.

### Concluding remarks

The results indicate that two C_4_ *Cleome* species, which have different Kranz anatomy and evolved independently in family Cleomaceae, have a similar developmental pathway for C_4_ during structural and biochemical maturation. The ontogenetic programme for this development involves control of cell-specific patterns of mitosis (rates and plane) and cell expansion, division of chloroplasts and mitochondria, structural differentiation and spatial distribution, cell wall modification, and plasmodesmata formation, with all of these characteristics occurring in close coordination with vascular system differentiation. The early partitioning of Rubisco, GDC, and PEPC expression between M and BS in both species shows that factors controlling their cell specificity must be activated as soon as the tissue become delimited. Further regulation occurs during development, with increased levels of expression of photosynthetic enzymes, and structural differentiation of M and BS cells and organelles. The results provide insight into stages to consider for transcriptome and proteomic studies to search for candidate genes which control structural and biochemical transitions in C_4_ development.

## Supplementary data

Supplementary data are available at *JXB* online.

Figure S1. Scanning electron microscopy of the abaxial surfaces of the young leaves of *Cleome angustifolia* (6mm) and *C. gynandra* (7mm) showing the level of stomata differentiation in the tip, middle, and base.

Supplementary Data

## Abbreviations:

BS: bundle sheath
GDC: glycine decarboxylase
M: mesophyll
M_ab_: abaxial mesophyll
M_ad_: adaxial mesophyll
NAD-ME: NAD-malic enzyme
PEPC: phosphoenolpyruvate carboxylase
*R*_d_: dark type respiration
Rubisco: ribulose 1,5-bisphosphate carboxylase-oxygenase
SM: spongy mesophyll
Γ: CO_2_ compensation point.
