# Supplementary material for: Differentiation of C4 photosynthesis along a leaf developmental gradient in two Cleome species having different forms of Kranz anatomy
Source: J Exp Bot. 2014 Feb 18;65(13):3525–41. doi: 10.1093/jxb/eru042 (PMC4085953; doi:10.1093/jxb/eru042)
Supplement: Supplementary Data [file supp_65_13_3525__index.html]

Differentiation of C4 photosynthesis along a leaf developmental gradient in two Cleome species having different forms of Kranz anatomy — Supplementary Data 

# Differentiation of C4 photosynthesis along a leaf developmental gradient in two *Cleome* species having different forms of Kranz anatomy

## Supplementary Data

Data files

**Files in this Data Supplement:**

- Supplementary Data - Supplementary Data
