# Supplementary material for: Differentiation of C4 photosynthesis along a leaf developmental gradient in two Cleome species having different forms of Kranz anatomy
Source: J Exp Bot. 2014 Feb 18;65(13):3525–41. doi: 10.1093/jxb/eru042 (PMC4085953; doi:10.1093/jxb/eru042)
Supplement: Supplementary Data [file supp_eru042_Fig_1_suppl.docx]

Differentiation of C_4_ photosynthesis along a leaf developmental gradient in two *Cleome* species having different forms of Kranz anatomy

**Nuria K. Koteyeva^1^, Elena V. Voznesenskaya^1^, Asaph B. Cousins^2^, and Gerald E. Edwards^2*^**

^1^Laboratory of Anatomy and Morphology, V. L. Komarov Botanical Institute of Russian Academy of Sciences, Prof. Popov Street 2, 197376, St. Petersburg, Russia. knouria@mail.ru, [elena-voz@](mailto:elena-voz@)mail.ru.

^2^ School of Biological Sciences, Washington State University, Pullman, WA 99164-4236 U.S.A. acousins@wsu.edu, edwardsg@wsu.edu

^*^To whom correspondence should be addressed. E-mail: edwardsg@wsu.edu

^*^Author for correspondence and reprint requests.

**Supplemental Material**

Figure S1 represents the scanning electron microscopy of the abaxial surfaces of the young leaves of *Cleome angustifolia* (6 mm) and *C. gynandra* (7 mm) showing the level of stomata differentiation in the tip, middle and base.


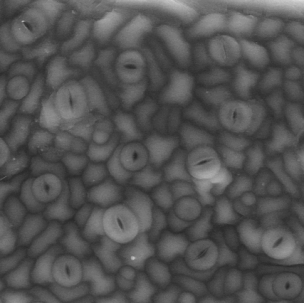

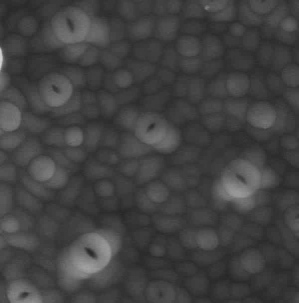

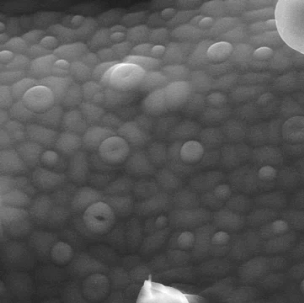

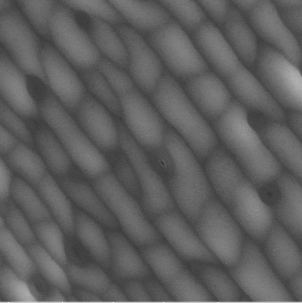

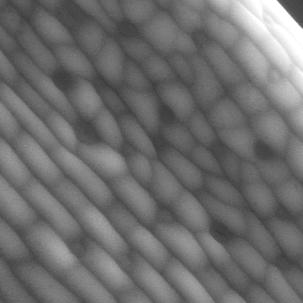

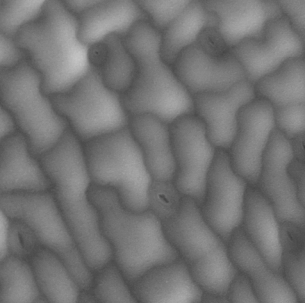

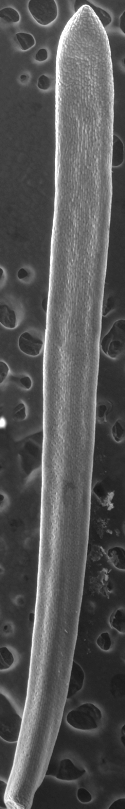

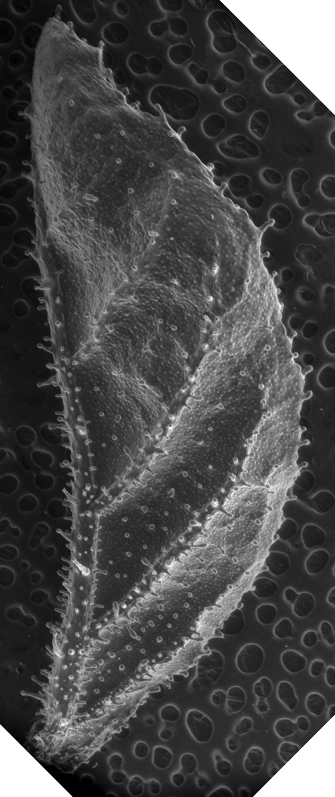


**A**

**B**

**C**

**D**

**E**

**F**

**G**

**H**

Fig. S1. SEM images of the abaxial surfaces of the young leaves of *Cleome angustifolia* (6 mm, A-D) and *C. gynandra* (7 mm, E-H) showing the level of stomata differentiation in the tip (B, F), middle (C, G) and base (D, H). Arrowheads point to the fully differentiated stomata with open pores and arrows point stomata in the process of differentiation.

The percentage of the stomata which are differentiated at the base, middle, and tip of young leaves was 8, 26 and 72%, respectively in *C. angustifolia* and 5, 33, and 69%, respectively in *C. gynandra.* Scale bars: 1 mm for A, E; 50 µm for B-D, F-H.
